# Tartary Buckwheat Starch Modified with Octenyl Succinic Anhydride for Stabilization of Pickering Nanoemulsions

**DOI:** 10.3390/foods12061126

**Published:** 2023-03-07

**Authors:** Jie Lin, Shasha Fan, Yuyue Ruan, Dingtao Wu, Ting Yang, Yichen Hu, Wei Li, Liang Zou

**Affiliations:** 1 State Key Laboratory of Southwestern Chinese Medicine Resources, School of Pharmacy, Chengdu University of Traditional Chinese Medicine, Chengdu 611137, China; 2 Key Laboratory of Coarse Cereal Processing, Ministry of Agriculture and Rural Affairs, Sichuan Engineering & Technology Research Center of Coarse Cereal Industrialization, School of Food and Biological Engineering, Chengdu University, Chengdu 610106, China; 3 School of Basic Medicine, Chengdu University, Chengdu 610106, China

**Keywords:** Tartary buckwheat starch, octenyl succinic anhydride, degrees of substitution, physicochemical property, emulsification stability

## Abstract

In this study, Tartary buckwheat starch was modified to different degrees of substitution (DS) with octenyl succinate anhydride (OS-TBS) in order to explore its potential for stabilizing Pickering nanoemulsions. OS-TBS was prepared by reacting Tartary buckwheat starch with 3, 5 or 7% (*w*/*v*) octenyl succinate in an alkaline aqueous solution at pH 8.5. Fourier-transform infrared spectroscopy gave peaks at 1726 cm^−1^ (C=O) and 1573 cm^−1^ (RCOO−), indicating the formation of OS-TBS. We further studied the physicochemical properties of the modified starch as well as its emulsification capacity. As the DS with octenyl succinate anhydride increased, the amylose content and gelatinization temperature of the OS-TBS decreased, while its solubility increased. In contrast to the original Tartary buckwheat starch, OS-TBS showed higher surface hydrophobicity, and its particles were more uniform in size and its emulsification stability was better. Higher DS with octenyl succinate led to better emulsification. OS-TBS efficiently stabilized O/W Pickering nanoemulsions and the average particle size of the emulsion was maintained at 300–400 nm for nanodroplets. Taken together, these results suggest that OS-TBS might serve as an excellent stabilizer for nanoscale Pickering emulsions. This study may suggest and expand the use of Tartary buckwheat starch in nanoscale Pickering emulsions in various industrial processes.

## 1. Introduction

Tartary buckwheat (*Fagopyrum tataricum,* (L.) Gaertn.) is a traditional edible and medicinal pseudo-cereal enriched with beneficial phytochemicals, including flavonoids, phenolics, steroids, fagopyrins, and d-chiro-inositol [1]. As a coarse cereal, starch constitutes about 70% of the mass of Tartary buckwheat seeds [2]. Tartary buckwheat is traditionally used to make noodles, breads, sakis and biscuits. Due to its high content of flavonoids and protein, Tartary buckwheat has become an important ingredient in the processing of functional foods in recent years. As a result, Tartary buckwheat has been extensively used in the food industry for the extraction of proteins or flavonoids, leaving Tartary buckwheat starch (TBS) as a by-product [3]. At present, large quantities of TBS are currently discarded or used as animal feed because of its untapped industrial value.

TBS has unique physicochemical properties, including a small granule diameter (almost 3–14 μm) [3], low starch solubility, high peak viscosity, strong hot and cold paste stability, low sensitization and low glycemic index [4]. The smaller granule size allows it to be used as for particle-based stabilization of emulsion, giving it a very wide range of potential applications in the food, pharmaceutical, cosmetic and other industries [5]. However, the surface properties and microstructure of native TBS particles may partially limit their application in certain areas. In order to improve the physicochemical properties of TBS, attempts have been made to modify it by physical and chemical methods such as repeated retrogradation, plasma treatment and the addition of polyphenols to reduce the digestibility or increase light transmission, swelling power and solubility of TBS [6,7,8].

Octenyl succinic anhydride (OSA)-modified starches, approved for food use by the US Food and Drug Administration in 1972, have been prepared from oat, quinoa, sago, maize, and wheat [9,10,11] and widely applied as a food emulsifier [12]. We hypothesized that it might be possible to improve the emulsifying properties of TBS by conjugating it with OSA. In other types of starch, the abundant OH groups along the starch backbone have been esterified with hydrophobic octenyl succinate to give rise to anionic starch containing COO [13], leading to better hydrophilicity and lipophilicity and therefore a higher emulsifying ability [14]. As a result of these advantages, OSA-modified starches from a variety of sources are being investigated for the stabilization of Pickering emulsions [15]. OSA-modified starch can contribute to Pickering emulsions containing bioactive substances such as lutein, β-carotene, curcumin, and resveratrol [16,17,18]. However, in almost all of these applications the starch-particle-stabilized droplets were limited to micrometric sizes. Numerous studies have demonstrated that reducing the size of dispersions to nanometric sizes can improve the stability and rheology of emulsions [19,20]. Therefore, it could be expected that the construction of Pickering nanoemulsions would be able to improve their efficacy and stability. Unfortunately, few studies have successfully demonstrated the preparation of Pickering nanoemulsions with droplet sizes below 500 nm [21].

The improvement of the surface properties of the starch granules and the choice of the appropriate physical treatment technique are essential for the formation of Pickering nanoemulsions. High-pressure homogenization (HPH) is a non-thermal physical process for reducing the particle size of a sample (emulsion or suspension) from the micron range to the nanometer range by using shear, impact and cavitation effects at high pressure [22]. Due to its high production efficiency and stability, HPH has been widely used to produce emulsions [23], nanosuspensions [24], liposomes [25], etc. However, few studies have focused on the application of HPH to produce Pickering nanoemulsions.

Therefore, we investigated the potential of OSA-modified TBS (hereafter, “OS-TBS”) as a stabilizer for Pickering nanoemulsions. In particular, we focused on the effect of the modification on the physicochemical properties of the starch granules and on the formulation and process of nanoemulsion preparation. The size, chain length, viscosities and thermal characteristics of starch influence their emulsifying characteristics [26,27]. Thus, in this study, OS-TBS with different degrees of substitution were prepared by modification with OSA, and then the morphology, physicochemical properties and emulsifying properties of the resulting formulations were systematically studied. The novelty of this study is the development of an OSA-modified small solid particle stabilizer based on TBS. Based on this, a new method for the preparation of nanoscale Pickering emulsions using HPH techniques was developed. This research will provide new information for future research and applications in starch modification.

## 2. Materials and Methods

### 2.1. Materials

Tartary buckwheat was obtained from the Key Laboratory of Coarse Cereal Processing at the Ministry of Agriculture and Rural Affairs at Chengdu University (Chengdu, China); 2-octenyl succinate anhydride (OSA, 95% pure), from Shanghai Maclean Biochemical (Shanghai, China); as well as amylose standard (96% pure) and amylopectin standard (87.2% pure), from Beijing Northern Weiye Institute of Metrology and Technology (Beijing, China); medium-chain triglycerides from Shanghai Yuanye Bio-Technology (Shanghai, China). All other chemicals in this study were analytical grade and purchased from Chengdu Kelong Chemical Company (Chengdu, China).

### 2.2. Isolation of TBS

TBS was isolated as described [3] with slight modifications. Briefly, Tartary buckwheat seeds were hulled, rinsed, crushed through a 100-mesh sieve, then sonicated in 80% ethanol at a ratio of 1:20 (*w*/*v*) for 30 min at 50 °C and 500 W in order to remove flavonoids and lipids. The precipitate was soaked for 24 h at room temperature in 0.3% NaOH solution at a ratio of 1:10 (*w*/*v*), then passed through gauze in order to further remove crude fibers and other impurities. The resulting starch slurry was centrifuged for 10 min at 4000 rpm; the supernatant and upper brown layer were discarded, and the remaining white layer was washed again with 0.3% NaOH. Centrifugation and washing with 0.3% NaOH were repeated three times. The final precipitate was dispersed in distilled water, neutralized to pH 7.0 by addition of 0.1 M HCl, washed with distilled water and centrifuged repeatedly until the supernatant was clear without brown layers and it formed a firm, stable white precipitate at the bottom of the tube. Finally, the precipitate was dried at 40 °C for 48 h, ground into a powder and passed through a 100-mesh sieve to eliminate agglomeration. The resulting starch was stored in a polyethylene bag at room temperature for later use.

### 2.3. Preparation of OS-TBS

OS-TBS was prepared using a method based on a previous report [28,29]. TBS powder (30%, *w*/*v*) was dispersed in distilled water with continuous stirring. OSA was diluted with anhydrous ethanol and dropped slowly into the TBS dispersion within 2 h while temperature was maintained at 35 °C. The pH of the starch slurry was adjusted to 8.5 with 3% NaOH solution and the esterification reaction was allowed to continue for 3 h at 35 °C. Then the pH of the starch slurry was adjusted to 7 using 1 M HCl. The slurries were centrifuged for 15 min at 4000× *g*, washed several times with distilled water, washed twice with 90% ethyl alcohol, dried at 45 °C for 24 h, passed through a 100-mesh sieve and stored in polyethylene bags at room temperature.

The different amounts of OSA (3, 5, and 7% based on the weight of TBS) were added to the TBS dispersion, and resulting slurries were denoted by OS-TBS-3, -5, -7, respectively.

### 2.4. Determination of the Degree of Substitution (DS) in OS-TBS

DS of OS-TBS samples was determined using a titration-based method [30]. Briefly, 0.5 g OS-TBS was stirred into 3 mL 2.5 M HCl for 30 min, then 10 mL 90% (*v*/*v*) isopropanol was added to each sample, stirring was continued for 10 min, and the mixture was centrifuged at 3000× *g* for 10 min. The sediment was washed thoroughly with 90% isopropanol until addition 0.1 M AgNO_3_ did not lead to appreciable formation of AgCl. The washed sediment was suspended in 30 mL of distilled water, heated in a boiling water bath for 30 min, and titrated with 0.1 M NaOH solution using phenolphthalein as the end-point indicator. *DS* was calculated using the formula [31]:

(1) $$DS=\frac{162\times \left(A\times M\right)/W}{1000-210\left(A\times M\right)/W}$$

where *A* was the titration volume of NaOH solution (mL), *M* was the molarity of NaOH solution, and *W* is the dry weight (g) of the OS-TBS. Native TBS served as the reference.

### 2.5. Amylose Content

The amylose content of TBS and OS-TBS was determined using an iodine-binding method [32]. Starch samples or standards were added to 90% dimethyl sulfoxide, incubated at 95 °C for 60 min, then reacted with 3.04 g/L iodine liquid dissolved in 90% dimethyl sulfoxide. Standard solutions of amylose and amylopectin were prepared in 90% dimethyl sulfoxide and used to create standard curves for amylose content based on absorbance at 510 nm and 620 nm. A solution of 90% dimethyl sulfoxide served as a blank control.

### 2.6. Physicochemical Properties of OS-TBS

#### 2.6.1. Fourier-Transform Infrared (FT-IR) Spectroscopy

The chemical structure of TBS and OS-TBS was analyzed qualitatively using a Spectrum Two FT-IR spectrometer (PerkinElmer, Boston, MA, USA). Samples were prepared by grinding the finely powdered starch with KBr (1:100, *w*/*w*) and spectra were obtained from 400 to 4000 cm^−1^ at a resolution of 4 cm^−1^ [33].

#### 2.6.2. Particle Size Distribution and Granule Morphology

The size of TBS and OS-TBS particles was analyzed using a Mastersizer 2000 (Malvern Instruments, Malvern, UK). Starch samples were suspended in distilled water over a range of light obscurations from 10 to 20%. Volume-averaged droplet size (D) was determined as described [3,4] by assuming refractive indices of 1.33 for water and 1.54 for starch [28,34].

Granules were observed under a scanning electron microscope (Hitachi Regulus 8100, Tokyo, Japan). Samples were mounted on double-sided adhesive tape on an aluminum stub, a layer of gold was sputtered on top, and the samples were imaged at an accelerating voltage of 15 kV. Images were taken at different magnifications (×2000 and ×5000) at a working distance (WD) of 14 mm to observe the dense structure of the particles and pores [35].

#### 2.6.3. X-ray Diffractometry (XRD)

The crystalline structure of TBS and OS-TBS was examined using an X-ray diffractometer (Bruker-AXS D8 Advance, Sigersdorf, Germany) operating at 40 kV and 40 mA with Cu-Kα radiation. Diffractograms were obtained over a range of diffraction angles (2 *θ*) from 5 to 40° at a rotational speed of 6.35 °/min [9]. Relative crystallinity (Rc), which indicates the proportion of the total diffractogram area that is crystalline, was calculated as described [36] using MDI-Jade software (version 6.0, Second Street, Livermore, CA, USA).

#### 2.6.4. Thermal Properties

The onset temperature (*To*), peak temperature (*Tp*), conclusion temperature (*Tc*) and gelatinization enthalpy (Δ*H*) were measured as described [37] using a Sirius DSC 3500 system (Netzsch Gerätebau, Selb, Germany). Starch (3 mg) and deionized water (triple mass) were weighed into an aluminum crucible, which was sealed and equilibrated at room temperature for 2 h, then heated from 30 to 130 °C at a rate of 10 °C/min. A sealed empty crucible served as the reference [38].

#### 2.6.5. Solubility Determination

The water solubility index (WSI) and swelling power (SP) of starch samples were determined as described [39]. The TBS and OS-TBS samples were dissolved at 2% (*w*/*v*) in deionized water and incubated for 30 min in a water bath at 50, 60, 70, 80 or 90 °C, then centrifuged at 4000 rpm for 15 min. The supernatant was recovered and dried to a constant weight at 105 °C. The WSI and SP were calculated using the following equations:

(2) $$WSI=\frac{W_{1}}{W_{0}}\times 100\%\;$$

(3) $$SP=\frac{W_{s}}{W_{0}\times \left(1-WSI\right)}\times 100\%\;$$

where *W*_0_ was the weight of starch; *W_s_*, the weight of the sediment after centrifugation; and *W*_1_, the weight of the supernatant after drying.

### 2.7. Contact Angle Measurement

Starch powder was compacted into a standard tablet 2 mm thick and the tablet was then immersed in medium-chain triglycerides. Then, 16 μL of deionized water was dripped lightly onto the surface of the tablet for 1 min and allowed to equilibrate. Three-phase contact angles were determined using a JY-82B device (Kruss, Hamburg, Germany) based on taking photos and the protractor method [40].

### 2.8. Preparation and Characterization of Pickering Emulsions

#### 2.8.1. Fabrication

The preparation of Pickering emulsion samples were determined as described [13,41] with slight modifications. OS-TBS particles (1–5 wt%) were fully dispersed in aqueous NaCl solution at various concentrations (0–100 mM), vortexed for 3 min to disperse the starch homogeneously, mixed with medium-chain triglycerides with or without Sudan III (10–50 vol%), homogenized at 200 bar for 1 min to form a coarse emulsion, then further homogenized at 1000 bar for 5 min using a high-pressure homogenizer (AH-Nano, ATS Engineering, Shanghai, China). The resulting Pickering emulsions were stored in capped, flat-bottom glass vials for further analysis (see below). The influence of pH (3–11) on Pickering emulsion stability was also investigated.

#### 2.8.2. Microstructure and Zeta Potential

The distribution of drop size in Pickering emulsions was observed using a TL3900CA optical microscope (Teelen, Shanghai, China) at an image magnification of 10 × 100. The emulsions were diluted 1:5 (*v*/*v*) in deionized water, and one drop of emulsion was poured onto the glass microscope slide. The distribution of drop sizes was also measured using a Zetasizer Nano 90 system (Malvern Instruments, Malvern, UK), based on refractive indices of 1.414 for medium-chain triglycerides and 1.33 for water [26]. The same device was used to determine zeta potential.

#### 2.8.3. Emulsification Index (EI) and Centrifugation Stability

An aliquot of emulsion (12 mL) was transferred to a 15 mL sample vial, sealed and stored at room temperature and photographed at 0, 15 and 30 days. The emulsification index (EI) of the corresponding Pickering emulsions were evaluated by the volume/height of the emulsified and precipitated layers after 1d and the EI was calculated as described [42] using the formula:

(4) $$\mathrm{EI}=\frac{H_{emulsion}}{H_{total}}$$

where *H_emulsion_* was the volume of the observed emulsion and *H_total_* was the sum of all the phases.

The stability of emulsions with centrifugation was investigated after centrifugation at 10,000× *g* for 10 min as described [43] using a 5810R refrigerated centrifuge (Eppendorf, Hamburg, Germany).

### 2.9. Statistical Analysis

All experiments were performed in triplicate, and data were reported as mean ± standard deviation. Data were analyzed statistically using one-way analysis of variance (ANOVA) in SPSS 25.0 (IBM, Chicago, IL, USA). Differences were considered significant if *p* < 0.05. Data plots were prepared using Origin 2021 software (OriginLab, Northampton, MA, USA).

## 3. Results and Discussion

### 3.1. Degree of Substitution (DS) and Amylose Content

Increasing the amount of OSA from 3 to 7% during the preparation of OSA-modified Tartary buckwheat starch increased the DS from 0.0184 to 0.0312 (Table 1), reflecting the greater availability of OSA groups to replace OH groups in the starch [10], while decreasing the amylose content from 9.61 to 7.73%. The negative correlation between DS and amylose content is consistent with the idea that starch esterification occurs primarily in the more accessible amorphous region, where amylose chains concentrate, rather than in the crystalline region [11,44]. The amylose content of TBS in our study was 12.11%, lower than the range from 19.63 to 25.63% previously reported for other TBS [45]. This discrepancy may reflect differences in the genetics and growth environment of the starch sources [46].

### 3.2. FT-IR Analysis

**Figure 1:**
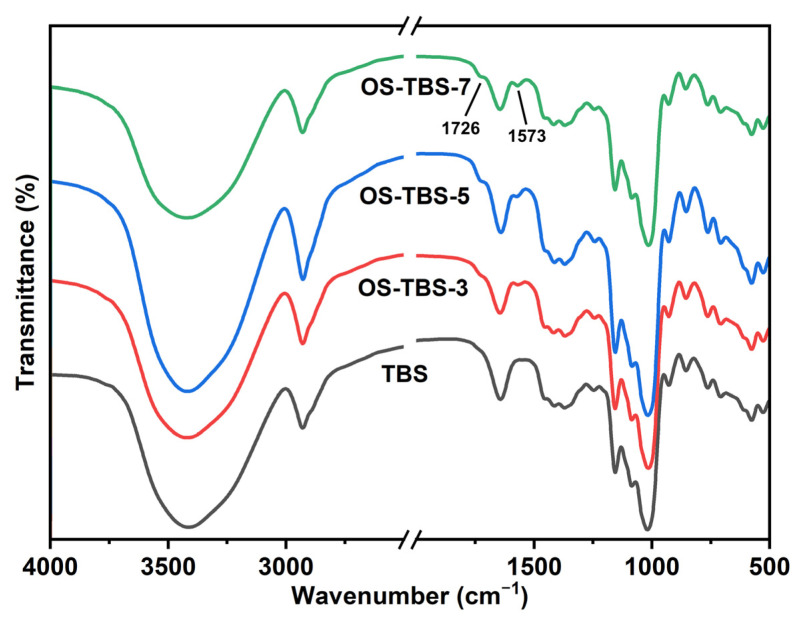
Fourier-transform infrared (FTIR) spectra (two peaks that appeared in the spectra of OS-TBS are indicated in the top spectrum) of TBS with and without OSA modification.

FT-IR analysis of TBS revealed strong O-H stretching vibration peaks at 3800–3000 cm^1^, tensile vibrations of C-H and bending vibrations of absorbed water at 2930–1640 cm^−1^ [42], and stretching of C-O bonds at 1200–800 cm^−1^ [14] (Figure 1). FT-IR analysis of OSA-TBS contained two additional peaks: one at 1726 cm^−1^, due to the C-O telescopic vibration of the ester carbonyl; and one at 1573 cm^−1^, due to asymmetric stretching of the RCOO vibration. These peaks were interpreted to indicate successful formation of OS-TBS because they were similar to previous reports [11,28]; they were consistent with the formation of the desired ester carbonyl group, and their area correlated positively with the DS.

### 3.3. Morphology and Particle Size Distribution

**Figure 2:**
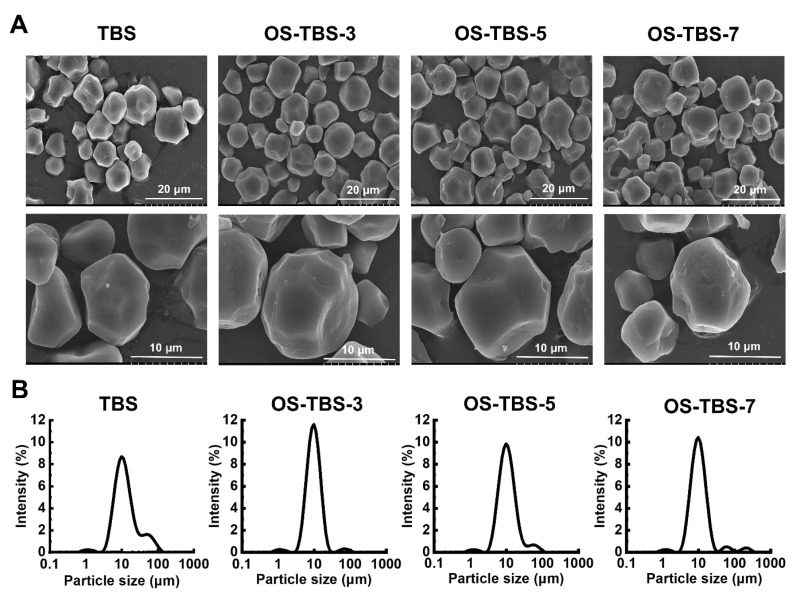
(**A**) Scanning electron micrographs (SEM) at different magnifications (×2000 and ×5000) and (**B**) particle size distribution of TBS and OS-TBS granules.

Scanning electron microscopy showed TBS granules to be irregular polygons with smooth, angular surfaces (Figure 2), similar to previous work [3]. The low water solubility of OSA limited the diffusion of OSA into starch and, therefore, the esterification of starch on the granule surface. Increasing the DS in OS-TBS particles increased surface erosion and abrasion; yet, most granules retained the native starch morphology (Figure 2A). While some studies have reported similar findings [12,28,47], other work has shown that OSA esterification created small surface pores in starch [40]. It is speculated that this may be related to the different sources of starch.

The volume-averaged droplet size was smaller for OS-TBS at all three DS (12.1–17.6 μm) than for the unmodified TBS (18.8 μm), (Figure 2B). Addition of NaOH during modification with OSA may lead to TBS granule shrinkage [47]. OS-TBS prepared with an OSA at 7% showed a bimodal distribution, suggesting greater aggregation of the hydrophobically modified starch granules than at lower DS [9].

### 3.4. XRD Analysis

**Figure 3:**
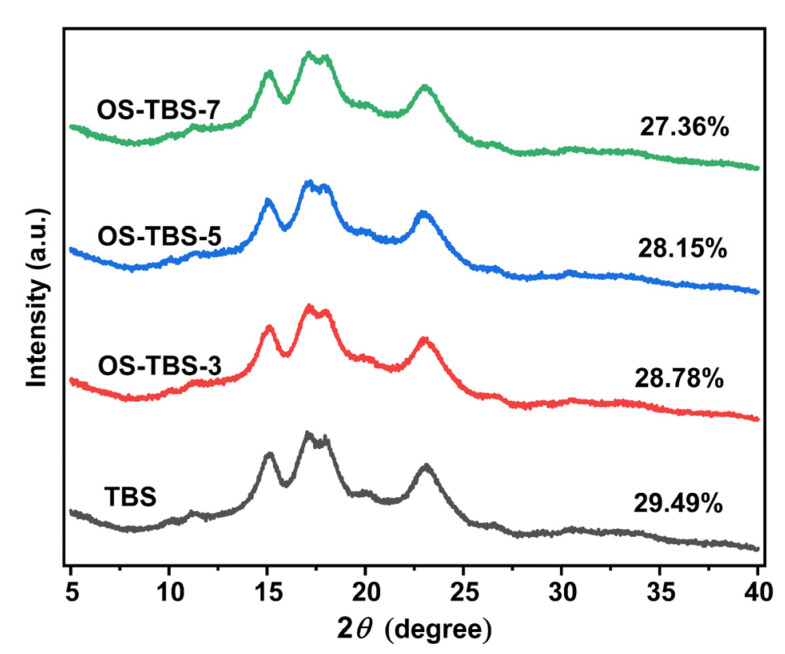
X-ray diffraction (XRD) patterns of TBS with and without OSA modification.

XRD analysis revealed TBS to have an A-type X-ray diffraction pattern with intense peaks at 2*θ* = 15, 17, 18 and 23° [3,48]. The crystallinity of TBS was 29.49% (Figure 3), consistent with previous work [3]. OSA esterification did not substantially change TBS crystal structure, although it did reduce crystallinity slightly. These results are consistent with the idea that OSA modification occurred primarily in amorphous regions of starch granules, with only a small number of OSA groups penetrating into the crystalline regions of the starch granules [12,42,49].

### 3.5. Thermal Properties

Differential scanning calorimetry of the heat changes in the starch during heating gelatinization indicated that esterification reduced *T_o_*, *T_p_*, *T_c_* and Δ*H* (Table 1). In fact, the effect of DS on the thermal stability parameters of the three modified samples was not significant, similar to previous work [50]. However, the introduction of bulky OSA groups reduces the integrity of the crystalline regions and the number of double helix structures in the starch granules, making the hydrogen bonds more prone to breakage during heating [9]. This results in a lower paste temperature and Δ*H*. This ultimately results in starch granules expanding at lower temperatures and reduces the energy required for pasting. In conclusion, our results suggest that OSA groups are present in the amorphous regions of TBS, where they may be predominant.

### 3.6. Water Solubility Index (WSI) and Swelling Power (SP)

WSI of TBS and OS-TBS increased with increasing temperature, particularly from 70 °C (Table 2), and it increased with DS. Sample solubility increased significantly when the temperature was higher than the conclusion temperature *T_c_*, based on WSI and thermal properties (Table 1), which may reflect the weakening of intermolecular hydrogen bonds by the introduction of OSA groups [49,51]. This increase in solubility may also improve viscosity, viscoelasticity, and emulsification [51,52].

### 3.7. Particle Wettability

The hydration capacity of starch is usually measured by SP. The SP of TBS before and after OSA treatment at different temperatures is shown in Table 2. As expected, OSA modification significantly increased SP compared to native TBS. Higher OSA additions (higher DS) resulted in higher starch swelling forces and the OS-TBS-7 had the highest SP (78%, 90 °C). The swelling behavior of the starch was related to the granule sizes and also closely associated with the fine structure of the starch [9]. OS-TBS is a small granular starch that usually has more amorphous regions and a branched starch structure. At high temperatures, the amorphous regions and the parts of the starch granules that are close to the crystalline regions become sticky and absorb excess water to form swollen granules. The OSA modification introduces hydrophobic carbon chains into the amorphous regions of the starch, weakening the strength of the internal hydrogen bonds which bind the starch molecules, thereby increasing SP [20].

**Figure 4:**
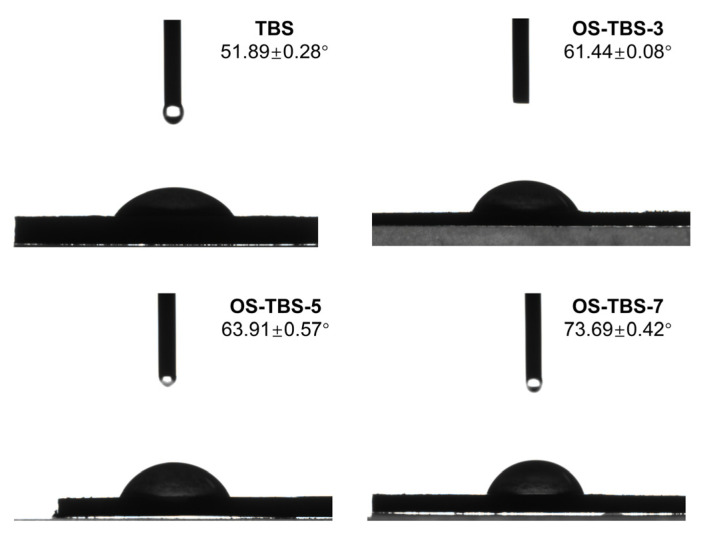
Contact angles of TBS with and without OSA modification.

The contact angle θ between the particle and the water phase was smaller than 90° not only for TBS but also for OS-TBS at different DS (Figure 4), suggesting that all these starches are more inclined to stabilize the O/W type of emulsions [53]. The contact angle increased from 61.5 to 71.3° as DS in OS-TBS increased, which is consistent with previous work [28,54] and can be attributed to the replacement of hydrophilic hydroxyl groups by the OSA group [14]. Thus, octenylsuccinylation can improve the hydrophobicity of starch granules and facilitate the adsorption of OS-TBS granules at the oil–water interface, implying usefulness as a good Pickering stabilizer.

### 3.8. Formation and the Storage Stability of OS-TBS-Based Pickering Emulsions

**Figure 5:**
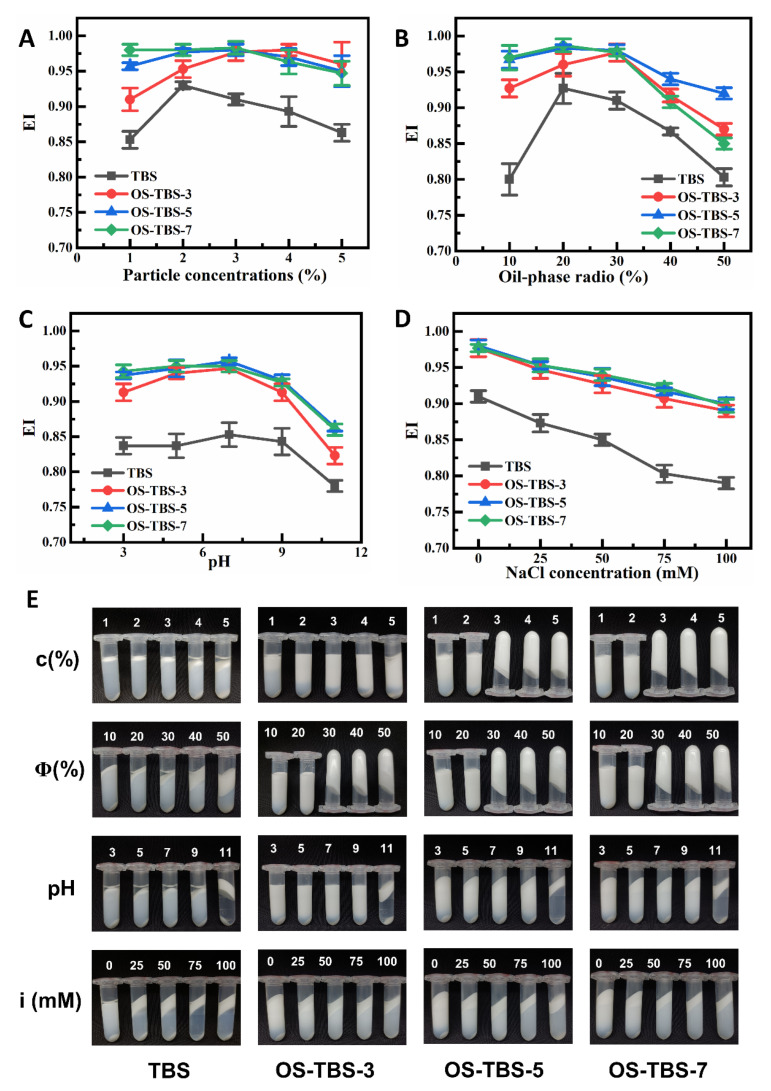
Effects of (**A**) TBS or OS-TBS concentration (c), (**B**) oil-phase volume fraction (Φ), (**C**) pH and (**D**) NaCl concentration (i) on the emulsification index (EI). (**E**) Photographs of Pickering emulsions stabilized by TBS or OS-TBS for the different conditions indicated, after centrifugation at 10,000× *g* for 10 min; (c) 20 vol% MCT, deionized water, without pH adjustment and salt free ions; (Φ) 4 wt% starch concentration, deionized water, without pH adjustment and salt free ions; (pH) 4 wt% starch concentration, 30 vol% MCT and salt free ions; (i) 4 wt% starch concentration, 30 vol% MCT, deionized water, without pH adjustment.

Whether Pickering emulsions were prepared with TBS or OS-TBS at different DS, the EI increased first and then decreased as stabilizer concentration increased at a constant oil-phase volume fraction of 20 vol% (Figure 5A). The EI was lower for Pickering emulsions prepared from TBS than for those prepared from OS-TBS, regardless of DS, oil-phase volume fraction, pH, or ionic strength (Figure 5B–D). Pickering emulsions prepared from the TBS group showed delamination after centrifugation, and emulsions containing 4–5 wt% concentrations showed extensive oil separation (Figure 5E). At the other extreme, OS-TBS-5 and OS-TBS-7 showed excellent stability when the concentration of stabilizer was >3 wt%. These observations likely reflect that increasing stabilizer concentration increases the viscosity of the continuous phase, resulting in the formation of an emulsion microgel on the surface of Pickering emulsions after centrifugation. We conclude that an OS-TBS concentration of 4 wt% may be optimal for stabilizing Pickering emulsions.

When the oil-phase volume fraction of TBS was 10 vol%, the surface coverage of oil emulsions was relatively low. It was better when the oil-phase volume fraction was 20%, when EI > 0.90. (Figure 5B). The EI of OS-TBS increased with the increasing oil-phase volume fraction (10–30 vol%). Further increases in the volume fraction actually decreased the coverage of starch particles, weakening the interfacial film and enlarging the droplet size, thereby destabilizing the emulsion. These results suggest an optimal oil-phase volume fraction of 30 vol%.

At pH 3.0–9.0, Pickering emulsions stabilized by TBS or OS-TBS showed good emulsion stability, and those based on OS-TBS showed an EI > 0.90 (Figure 5C). At pH 11, phase separation occurred in all cases (Figure 5C,E), which was consistent with previous work [55] and may reflect alkaline degradation of starch granules. These results suggest that OS-TBS stabilizes Pickering emulsions best at pH 7.0.

Finally, we identified lower ionic strength as best for OS-TBS to stabilize Pickering emulsions because increasing the ionic strength from 25 to 100 mM destabilized the emulsions, increasing emulsion particle size, particle flocculation and sedimentation (Figure 5D,E). These results suggest that salt ions can induce electrostatic shielding of droplet charges and possible aggregation. Therefore, we do not recommend the addition of salt ions when using OS-TBS for the preparation of Pickering emulsions.

**Figure 6:**
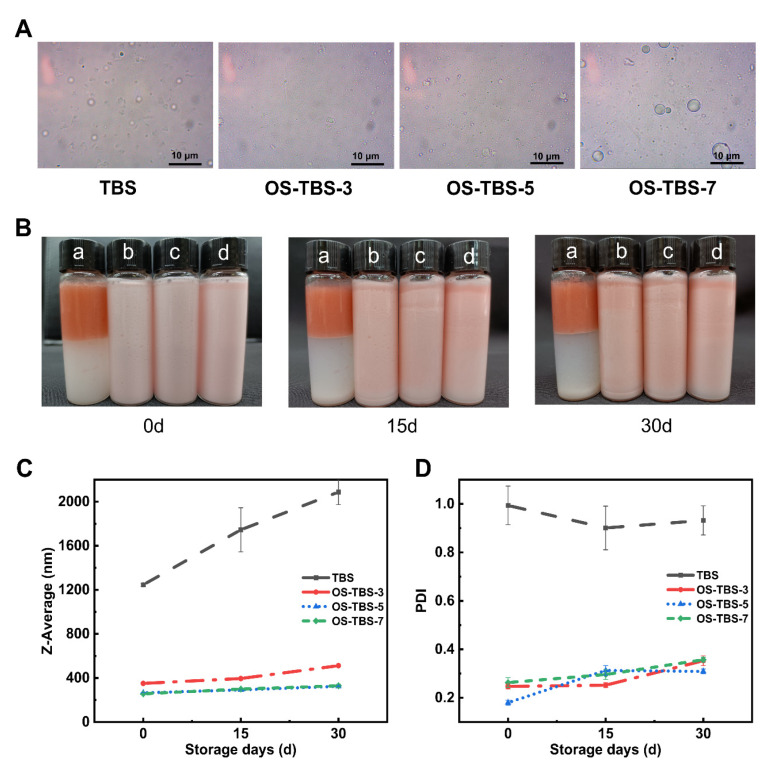
(**A**) Micrographs of Pickering emulsions stabilized by TBS or OS-TBS under optimal conditions (4 wt% starch concentration, 30 vol% MCT, no salt ions and pH is neutral.) on day 0. (**B**) Photographs of Pickering emulsions stabilized by (a) TBS, (b) OS-TBS-3, (c) OS-TBS-5, or (d) OS-TBS-7 after the indicated storage times. (**C**,**D**) Particle size and polydispersity index (PDI) of the emulsions during storage.

The distribution of droplet size in Pickering emulsions is shown in (Figure 6A); it can be seen that the droplet sizes in the OS-TBS groups were smaller and more uniform compared to the TBS group. During storage at 25 ± 0.5 °C for 30 days, the TBS group was stratified significantly, while the OS-TBS groups remained uniform and stable without delamination occurring (Figure 6B). In addition, the droplet size and polydispersity index (PDI) of the TBS group increased substantially; the PDI was close to 1. The OS-TBS groups, in contrast, remained essentially constant. (Figure 6C,D). The initial z-average of our OS-TBS groups, ~250 nm, was much smaller than the value reported in previous work [11,54], which may reflect that we used a high-pressure homogenizer. The resulting nanoscale emulsions may be stabler than those prepared with a high-shear mixer, and they may lead to good stability and better bioaccessibility of the encapsulated compounds.

## 4. Conclusions

This study investigated the changes in the crystalline structure, SP and emulsifying ability of TBS modified by different percentages (3, 5 and 7%) of OSA. The OSA modification slightly increased the particle size distribution of TBS and reduced the apparent straight-chain starch content. XRD patterns showed that the OSA modification occurred mainly in the amorphous region and had less effect on the crystalline region, which is consistent with the OSA treatment maintaining the morphological results of the starch, which are consistent with the OSA treatment maintaining the shape and integrity of the granules. We also investigated the effect of various factors on the emulsification properties of Pickering emulsions prepared by the HPH method using OS-TBS as a stabilizer. Our results suggest that Pickering nanoemulsions can be maximally stabilized by OS-TBS when the HPH method is used with a stabilizer concentration of 4 wt%, an oil phase volume fraction of 30 vol%, minimal ionic strength and neutral pH. The higher DS in OS-TBS result in smaller and more stable emulsified particles. Although the effects of various factors (e.g., DS, ratio of straight-chain to branched starch) on the physicochemical properties of OSA starch have been extensively investigated, this study further demonstrates the synergistic effects of OSA treatment, small particle-size characteristics and HPH methods in the preparation of Pickering nanoemulsions. Our work provides the first demonstration that a combined strategy offers the unique advantage of significantly improving the properties of TBS and lead to stable Pickering nanoemulsions for various industrial applications.

## Figures and Tables

**Table 1:** Amylose content, degree of substitution (DS) and thermal properties of TBS and OS-TBS.

Sample	Amylose Content (%)	DS	*T_o_* (°C)	*T_p_* (°C)	*T_c_* (°C)	Δ*H* (J/g)
TBS	12.11 ± 0.60 ^a^	NA	61.90 ± 0.08 ^a^	66.63 ± 0.09 ^a^	72.40 ± 0.68 ^a^	11.89 ± 0.99 ^a^
OS-TBS-3	9.61 ± 0.36 ^b^	0.0184 ± 0.0017 ^c^	60.83 ± 0.17 ^b^	65.57 ± 0.12 ^b^	71.47 ± 0.24 ^b^	8.90 ± 1.24 ^a^
OS-TBS-5	8.48 ± 0.13 ^c^	0.0251 ± 0.0010 ^b^	60.37 ± 0.19 ^c^	64.77 ± 0.05 ^c^	71.37 ± 0.19 ^b^	8.71 ± 0.22 ^a^
OS-TBS-7	7.73 ± 0.10 ^d^	0.0312 ± 0.0007 ^a^	58.83 ± 0.23 ^d^	63.47 ± 0.32 ^d^	70.63 ± 0.20 ^b^	8.79 ± 0.17 ^a^

Values are mean ± standard deviation. Different letters within a column indicate significant differences (*p* < 0.05).

**Table 2:** The water solubility index (%) and swelling power (%) of TBS and OS-TBS.

Parameter	Sample	Temperature (°C)				
		50 °C	60 °C	70 °C	80 °C	90 °C
WSI (%)	TBS	0.91 ± 0.01 ^d^	1.06 ± 0.01 ^d^	4.75 ± 0.27 ^d^	7.20 ± 0.37 ^d^	9.99 ± 0.53 ^d^
	OS-TBS-3	1.06 ± 0.01 ^c^	2.03 ± 0.11 ^c^	8.18 ± 0.08 ^c^	31.36 ± 1.55 ^c^	76.55 ± 1.19 ^c^
	OS-TBS-5	1.52 ± 0.07 ^b^	5.73 ± 0.81 ^b^	26.66 ± 0.80 ^b^	50.81 ± 1.88 ^b^	78.37 ± 1.89 ^b^
	OS-TBS-7	1.91 ± 0.31 ^a^	8.39 ± 0.58 ^a^	53.53 ± 0.48 ^a^	65.75 ± 0.50 ^a^	80.50 ± 0.31 ^a^
SP (%)	TBS	3.55 ± 0.89 ^c^	2.47 ± 0.11 ^d^	7.39 ± 0.07 ^d^	8.49 ± 0.42 ^d^	8.53 ± 0.29 ^d^
	OS-TBS-3	3.55 ± 0.84 ^c^	4.42 ± 0.27 ^c^	10.70 ± 0.65 ^c^	14.19 ± 1.18 ^c^	38.72 ± 0.30 ^c^
	OS-TBS-5	3.83 ± 0.25 ^b^	5.82 ± 1.16 ^b^	22.12 ± 0.82 ^b^	31.48 ± 2.8 ^b^	52.84 ± 5.15 ^b^
	OS-TBS-7	4.29 ± 0.41 ^a^	10.36 ± 0.25 ^a^	36.18 ± 2.97 ^a^	52.41 ± 4.49 ^a^	78.63 ± 2.44 ^a^

Values shown are means ± standard deviations. Different letters within a column indicate significant differences (*p* < 0.05).

## Data Availability

The data presented in this study are available on request from the corresponding author.
